# Patient-, care partner-, and clinician-proposed solutions to address the time toxicity of cancer care

**DOI:** 10.1007/s00520-025-09954-0

**Published:** 2025-10-18

**Authors:** Whitney V. Johnson, Sai S. Valisekka, Obafemi O. Ogunleye, Samuel X. Stevens, Manju George, Allison Breininger, Michael Anne Kyle, Christopher M. Booth, Timothy P. Hanna, Rachel I. Vogel, Helen M. Parsons, Anne H. Blaes, Arjun Gupta

**Affiliations:** 1 University of Minnesota, MN Minneapolis, USA; 2 School of Medicine, The University of Sydney, Camperdown, Australia NSW; 3 Paltown Development Foundation/COLONTOWN, Edgewater, MD USA; 4 Cancer Center and Research Institute, University of Mississippi Medical Center, Jackson, USA MS; 5 John D Bower School of Population Health, University of Mississippi Medical Center, Jackson, USA MS; 6 The Negative Space, Minneapolis, USA MN; 7 University of Pennsylvania, Department of Medical Ethics and Health Policy, Philadelphia, USA PA; 8 Sinclair Cancer Research Institute, Division of Cancer Care and Epidemiology, Kingston, ON Canada; 9 University of Minnesota, Division of Hematology, Oncology, and Transplantation, Minneapolis, USA 516 Delaware Street SE, MMC 480, PWB 14-100, MN 55455

**Keywords:** Time burdens, Time toxicity, Cancer, Care delivery, Health system navigation, Quality of life, Care partners

## Abstract

**Purpose:**

As the concept of time toxicity has gained traction in the oncology community, work has largely focused on mapping and measuring time burdens. This qualitative study sought to elicit perspectives from people with lived or professional experience with cancer care on strategies to decrease the time burden of oncology care.

**Methods:**

We conducted semi-structured interviews with 47 participants (16 patients with advanced gastrointestinal cancer, 15 of informal care partners, and 16 oncology clinicians with diverse roles) from a single academic cancer center in Minneapolis, Minnesota. Interviews were conducted between February 2023 and October 2023 and transcribed and analyzed using a hybrid approach.

**Results:**

Five key themes emerged as solutions to address time toxicity: (1) tailored ambulatory scheduling based on patient and care partner needs; (2) improved administrative and logistical support; (3) home-based care when safe, feasible, and preferred; (4) transparent and empathetic communication of time demands, and (5) innovative care models and delivery. Participants emphasized that the threshold between helpful and burdensome care in the context of a cancer diagnosis differs for each person, requiring individualized solutions.

**Conclusion:**

Findings from this study will aid the oncology community in considering and designing interventions to decrease the time burdens of cancer care on patients and their care partners. Implementing changes to meet the need for individualized, person-centered interventions, focusing on individuals’ preferences and unique circumstances, will require health system will and motivation .

**Supplementary Information:**

The online version contains supplementary material available at 10.1007/s00520-025-09954-0.

## Introduction

Since 2022, when the term “time toxicity’’ was first proposed to name and bring to attention the time-related burdens of care [1], the concept has gained momentum with multiple qualitative and quantitative studies assessing and describing the issue [2–10]. These have helped identify, map, and measure the time-related burdens of care. Work has identified that patients with advanced cancers spend approximately 1 in 5 days with in-person health care contact [11], while typically surviving for months or a few years. Seemingly-short ambulatory appointments can turn into all-day affairs [3], and that even when home, many medical tasks and logistic and administrative burdens take up patients’ and care partners’ time [2, 12, 13]. As the field continues to advance with an increasingly sophisticated understanding of time toxicity, a key clinical question relevant to all oncology stakeholders—patients, care partners, and clinicians as well as health systems, payers, and policymakers—is how to ameliorate the time burdens of cancer care.

Current efforts to address time burdens are limited to pilot studies [14], or are largely a fortunate byproduct of necessary care changes implemented over the COVID19 pandemic [15–18], with retrospectively realized benefits related to time toxicity. However, broad perspectives from frontline oncology stakeholders—people with lived or professional experience with cancer—are essential to craft sustainable and scalable interventions. Some amount of time spent in health care is necessary, and support from clinicians during visits valued by patients and care partners, especially for sick persons with advanced stage cancer; blindly cutting visits may actually be detrimental [19]. Additionally, patients and care partners may have diverse and divergent views on how best to address time toxicity—optimizing for one may burden the other, and vice versa [2]. Clinician input is additionally critical to understand their perspective and what might be immediately actionable from the health systems’ side. Thus, there is a critical need for broad multi-stakeholder perspectives on how best to address time toxicity. Gastrointestinal cancers account for over a quarter of global cancer cases and over a third of cancer deaths [20]. Patients with advanced stage gastrointestinal cancer have high rates of time toxicity [21, 22] and experience care in multiple settings (ambulatory, facility-based, and home-based care). The informal care partners of these patients also face significant time burdens [23]. These patients and care partners thus form an ideal population in which to study potential solutions to reduce time toxicity.

We thus sought to elicit the perspectives of patients with advanced stage gastrointestinal cancer, their informal care partners, and clinicians on solutions to address time toxicity.

## Methods

### Participants, recruitment, and setting

This qualitative study was conducted at the M Health Fairview clinics affiliated with the University of Minnesota, a National Cancer Institute (NCI)—Designated Cancer Center. Between February and October 2023, we employed purposive and criterion sampling techniques to recruit three stakeholder groups: adult patients with advanced gastrointestinal cancers receiving systemic therapy; their informal (unpaid) care partners; and oncology clinicians (including physicians, advanced practice providers, nurses, social workers, and schedulers). The study design intentionally sought diversity across gastrointestinal cancer types, participant ages, genders, and clinical roles. The study received institutional review board approval and followed the Consolidated Criteria for Reporting Qualitative Research (COREQ) reporting guidelines (Supplementary Table 1).

#### Data collection

We conducted semi-structured qualitative interviews in two phases: a pilot phase (February–March 2023; *n* = 9, with three participants from each stakeholder group) and a main phase (May–October 2023; *n* = 38). An interdisciplinary team, including advocates, qualitative research experts, and oncologists, used the pilot interviews to further refine the interview guide and incorporate participant feedback. Interviews lasted approximately 45 min and were conducted via video conference, telephone, or in-person according to participant preference. The interview guide explored perceptions of time-consuming aspects of cancer care, associated burdens, consequences, and proposed solutions (Supplementary Table 2). All interviews were audio-recorded, anonymized, and transcribed verbatim. Patient and care partner participants were offered modest compensation ($25 gift cards).

### Analysis

We applied thematic analysis using a hybrid approach incorporating inductive (data-driven) and deductive (theory-driven) coding methods. During the pilot phase, codes were developed de-novo using inductive analysis, creating an initial code book and themes. Subsequently, we used deductive coding during the main phase of interviews. Two researchers independently coded transcripts using NVivo software (version 12), then comparing their codes to merge them into a shared codebook. The coders would subsequently meet to discuss codes, making revisions and clarifying themes as needed until consensus was reached. The analysis process began concurrently with data collection in February 2023 and continued through February 2024.

## Results

### Participant characteristics

We interviewed a total of 47 participants (16 patients, 15 care partners, and 15 clinicians). Seventy-five percent of patients were women, 69% were white, and half were < 60 years old (Table 1). Interviewed care partners were younger than patients (53% less than 46 years old), 60% female, and 73% white. Among clinicians, physicians (7), advanced practice providers (2), nurses (4), social workers (4), and clinic schedulers (1) were represented. Three clinicians brought more than 20 years of experience, while three were in their first 5 years of practice. A prior manuscript has presented the sources of, persons impacted, and consequences of time burdens [10]. The current manuscript covers proposed solutions.

**Table 1:** Participant characteristics

Characteristic	Patients (*n* = 16), no. (%)	Care partners (*n* = 15), no. (%)	Clinicians (*n* = 16), no. (%)
***Age group***			
18–45 years	2 (12.5)	8 (53.3)	
46–60 years	6 (37.5)	4 (26.7)	
61–75 years	7 (43.8)	3 (20.0)	
76 years or older	1 (6.2)	0 (0.0)	
***Gender***			
Woman	12 (75.0)	9 (60.0)	11 (68.7)
Man	4 (25.0)	6 (40.0)	5 (31.2)
***Racial background***			
Asian	3 (18.7)	2 (13.3)	
Black or African American	2 (12.5)	2 (13.3)	
White	10 (62.5)	11 (73.3)	
Not disclosed	1 (6.2)	0 (0.0)	
***Annual household income***			
< $40,000	4 (25.0)	2 (13.3)	
$40,000–$79,999	6 (37.5)	7 (46.7)	
≥ $80,000	4 (25.0)	6 (40.0)	
Not reported	2 (12.5)	0 (0.0)	
***Clinical role***			
Physician			7 (43.8)
Nurse			4 (25.0)
Advanced practice provider			2 (12.5)
Scheduler or administrative staff			1 (6.2)
Social work or psychosocial support			2 (12.5)
***Years of professional experience***			
Less than 5 years			3 (18.7)
5–10 years			5 (31.2)
11–20 years			5 (31.2)
More than 20 years			3 (18.7)

### Themes

We grouped the major findings from the qualitative interviews into five major themes with 24 subthemes (in parentheses in each section) highlighting proposed solutions to the time toxicity of cancer care presented here, and in Table 2.

**Table 2:** Themes, subthemes, and illustrative quotations

**Theme 1: Tailored ambulatory scheduling based on patient and care partner needs**	
**Subthemes**	**Illustrative quotations**
Ambulatory care close to home	"*I was going over to [the university hospital] and then they got things set up at [community site] which is just five minutes from me, which made it much more convenient and easier to do." (Patient 3)* *"And helping the patients who live far away get what they can close to home, even if it's just a few things, if it's a couple of days a couple of times in a month that they don't have to drive all the way here, and we find a way to partner with a local institution and make their lives just 2% easier." (Clinician 10)*
Aligning appointments with patient/care partner availability	*"Everybody likes to come in at different times. I like to go really early. […] We like to get down there and get back home and sometimes it doesn't work out that way. But if possible, that works best for us." (Patient 1)* *"The system kicks in and just says, these are the days we want you to come. They don't ask me,* *‘What days are good for you to come in?*’* It would be a lot easier if I could go, *'*Well, Tuesdays are my days off. So, can I make your appointments on Tuesdays to get the chemo? That way, I won't lose that day.’ […] That would help a lot.*”* (Patient 14)*
Clustering appointments on the same day	*“She was able to go in, get her labs, see her doctor […] and do the infusion on the same day so it was only one trip instead of multiple times during the week, so that was very helpful.” (Care Partner 14)* *"Sometimes my scheduler will, with my approval, ask if it's okay to see patients while they are in the infusion center if it works better with their schedule, and sometimes that is a little bit faster for them than having to formally see me in clinic because the only clinic spot is the day before." (Clinician 12)*
Taking patient preference into account	*‘’I just separated two therapies from back-to-back on the same day into two separate days. The two-in-one day was too much for my [relation], however having them on two separate days is definitely more time consuming for me.’’ (Care Partner 6)* *‘’It is difficult to get all your appointments to line up one after the other. It’s actually easier for me sometimes to hop in for appointments and I don’t have to miss work that day.’’ (Patient 8)*
Designated schedulers	*"[Health system] has always just called me— in fact, it’s been [scheduler name] almost every time. He told me what needs to be done, by when, and helped set things up. […] There’s a choice of where I want to go. So it has been very, very little burden. That's the way it should be for everybody." (Patient 2)* *“There should be one person assigned to my [relation] and me, so that’s the person that is going to be there to answer the questions for us and not be round-about. […] So having to explain, my [relation] is dying, to 30 different people, this is what I’m trying to schedule, can you help me out with it, [and then to hear] no I can’t, let’s pass you to this person, and then explain it again. That has been such a waste of my time that I just get so fed up with it I just give up.” (Care Partner 1)*
Ensuring only necessary and carefully prepared appointments	*‘’We go for labs and there’s a wait. There’s no labs ordered. We are just sitting there while they check on their computer, they call people. And we are sitting there waiting.’’ (Patient 2)* *"So sometimes before appointments, I check up patients, and the scan we were going to review, it did not happen. So I try to cancel their visit, so they don’t come in, you know, for nothing. But it's hard to sometimes get in touch with the patient or say don't come in tomorrow. We don't have what you need. I think that we could do a better job." (Clinician 13)*
**Theme 2: Improved administrative and logistical support**	
**Subthemes**	**Illustrative quotations**
Assistance with health insurance and costs	*"I was at the pharmacy and insurance did not cover that medicine. It was difficult to reach them, to get the insurance involved. But then somebody helped me use the online app and they showed me what to do. […] I would have been stuck there with no idea of what to do." (Patient 9)* *" A lot of times a patient will ask me, ‘oh my infusion cost $2000, who do I call?’ […] If we were able to streamline getting insurance coordinated, all of that could be very helpful to them." (Clinician 3)*
Assistance with other administrative and logistical tasks	*‘’[volunteer organization] helped me apply for the [foundation grant]. Those $1000 were a life-saver then. I had spent so much time just worrying and looking around. I don’t think I would have figured it out on my own.’’ (Patient 12)* *‘’If they drop [FMLA paperwork] off at the front desk, we need to call them. We need to talk to the patient when we receive blank paperwork, because we need to know what date they want. And sometimes they want to continue working. They just want to continue working at like a part time status. We don’t really know what they want, so we need to talk to them regardless. It’s a lot of work, but I think it really helps them.’’ (Clinician 14)*
Administrative support for clinicians	*“We feel bad, they’ve got all these things to deal with medically and then we’re tapping them on the shoulder going, ‘can you sign my little piece of paperwork here again?’ It's like, you should be saving lives, not signing off on our little stuff.” (Care Partner 7)* *"The bureaucracies of insurance and all of the time that it takes to get people what they need, is a huge stressor for not only patients and families, but also for the staff that have to do it. I can't tell you how many hours I spent on hold, trying to get authorizations or patient's medications, to get to a real person to try to get the authorization to move them to a different level of care." (Clinician 14)*
Current staffing levels are inadequate	*“They need to find more care coordinators to really help co-design the care, the plan, with the doctors. And its about having aware care coordinators that walk through the entire process with you and your [relationship].” (Care Partner 3)* *"So, there's only so many social workers. And there's way more patients. And so staffing in health arenas, regardless of if it's an academic hospital, all the way into the community house, or a nursing home facility based care, the staffing universally, is poor." (Clinician 15)*
**Theme 3: Home-based care when safe, feasible, and preferred**	
**Subthemes**	**Illustrative quotations**
Telemedicine appointments	*"The phone call or virtual visit is very nice. I understand that once in a while you do have to go in and have an office visit, but if it's just a check in, it's really nice to have those options. […] I've saved a lot of time." (Patient 3)* *"They have offered virtual visits and I think especially for those of us that live far away, it's very nice to have those virtual options." (Patient 7)*
Formal home-based health services	*"I'm doing it [IV fluids] in the comfort of my house where mentally and psychologically, there's a comfort level already. I can be in my home." (Patient 10)* *“My goodness. Not having to come in [and get pump disconnect at home] was excellent. She felt crappy on the third day after chemo, and then not to have to drive an hour and go back in.” (Care partner 7)*
Policy reform and systemic support for home-based care	*"If your insurance would pay, you could get the shots and do it at home. But if your insurance wouldn't pay, you would have to go in for those shots which is really, really annoying." (Patient 8)* *"A lot of times insurance says no [to covering home care], unless they are homebound. I don't know what the reason is for that because it makes no sense, it makes their life so much less stressful in terms of just traveling." (Clinician 4)*
**Theme 4: Transparent and emphathetic communication of time demands**	
**Subthemes**	**Illustrative quotations**
Information and guidance from oncology teams	*‘’We rely on our PCP, who is great, to try to interpret, separate the wheat from the chaff. We ask her, ‘’What is this appointment, and what is this treatment.’’ Specialists are so in their lane they don't see the whole person.’’ (Care Partner 6)* *“Well, for me, I think it would be helpful just from a planner point of view. Having some sort of education about what to expect from the oncologist or the nurse or the social worker… to be realistic about the process and what things are going to look like.” (Care Partner 13)*
Clear expectations about expected time burdens	*“I would say transparency around wait times. I really hate it when the doctor says "it’ll be quick" and then you go to book with the receptionist and they book a 3-h infusion, and then it actually takes 5 h.” (Care Partner 13)* *“What seems routine to the provider is not routine for us. And so, not having complete knowledge or not giving a full understanding of what the expectations are creates a lot of burden. Knowing what to expect firsthand and giving more realistic times, that’s beneficial.” (Care Partner 13)*
Clinical teams showing interest in addressing time burdens	*“I can't tell you how transformative it would be for oncology teams to say, ‘Okay, I want you to keep a log of the time you're spending and then let's check it in a month.’ Then to assess, how much time is being spent where, and are there social services or social workers who can help address A, B, or C. So it's not just about managing your treatment and medications, but making sure you can continue to participate in the world and receive this support you need for living well and independently.” (Care Partner 12)* *‘’It was months before anyone ever asked me how I was, and that was my neighbor, who had also been a caregiver. I think oncologists even saying, "This must be terribly hard’’— and that applies to time as well. I think that would help a lot.’’ (Care Partner 9)*
Communicating about time burdens using objective measures	*"[Contact days] make a ton of sense because [a visit] is maybe only an hour but it shoots your whole day because you still have to make plans and adjust your entire schedule. I would like that information." (Patient 6)* *"I think it'd be a really beneficial way to pose it to them. […] I actually had a patient just two days ago say, ‘I'm not coming in on that day, that's my only home day.’" (Clinician 3)* *"We have an obligation as healthcare providers to educate our patients on their level, so they can understand it. And I think that means breaking it down to a dichotomous lost day/home day structure." (Clinician 10)*
Informed decision-making around time-intensive treatments	*‘’If given a choice, I would choose a shorter time with higher quality of life. I mean, there are a lot of things that go along with that, too, I’m sure. There’s saving money; there’s not having the burden of travel; all that sort of thing would make a huge difference, yes. I think that would be a really important piece of information a doctor really should be required to give somebody. (Patient 8)* *‘’As I tell patients, cancer will take everything it can from you, and more. It is critical to take treatment breaks, go to that wedding, go to that cruise. And I think time toxicity can help get that message across.’’ (Clinician 9)*
Availability of objective information about time burdens	*"I think education on a larger scale for physicians and other health care providers about time toxicity would be helpful. I mean its obvious, but If you're not thinking about an issue and it's not at the forefront, then you're less likely to make small changes that would make a meaningful difference for your patients." (Clinician 12)*
**Theme 5: Innovations in care models and delivery**	
**Subthemes**	**Illustrative quotes**
Shorter treatment courses	*"I was scheduled to have chemo for six months and after this trial, they showed that you could have chemo for three months and that was just as good." (Patient 9)*
Oral treatment options	*"I was on the FOLFIRINOX chemo, and then after that was done, my oncologist told me I could do the [brand-name oral chemotherapy], and that was even mailed to my house, so that changed my life, there wasn't even a burden of going to the clinic to get anything, which was helpful." (Patient 2)*
Less frequent treatment schedules	*‘’I’ve wanted to change someone’s pembro, its an immunotherapy, to every 6 weeks, instead of every 3 weeks, but you know, the insurance has said no. Its so frustrating. The guidelines say every 6 weeks is ok. And everyone would benefit.’’ (Clinician 6)*
Faster formulations	*"That drug shifted from an IV to having a new option of subcutaneous with a five-minute injection and talk about an improvement in quality of life. […] I got hours of my day back because of that transition from IV to subcutaneous. (Patient 5)*
Digital communication technologies for clinicians	*‘’Now that we have digital [software name], I often upload the document and then type in it or copy and paste from the chart so that I can get it digitally completed. It really is so much nicer, yes, I can get back to patients quicker.’’ (Clinician 14)*

### Theme 1: Tailored ambulatory scheduling based on patient and care partner needs

Across six subthemes (1–6), participants emphasized that scheduling ambulatory appointments around patients’ and care partners’ lives and preferences, when clinically appropriate, is critical in decreasing time burdens. Patients noted (1) receiving ambulatory care close to home made treatment “much more bearable.” Receipt of care “just five minutes from me,” compared to long travel times to the initial clinic they were assigned to, made a significant difference in reducing burdens related to cancer care. Clinicians noted successful models, such as the Veterans’ Health Affairs system, which often outsources care to facilities closer to patients.

Participants noted that (2) aligning appointments with patient and care partner availability was critical for time management and quality of life, and overall preferred (3) clustering multiple ambulatory appointments on the same day, instead of multiple trips to the clinic. However, (4) taking individual patient preference into account when scheduling is critical as participants did not universally prefer clustering appointments on the same day: two patients highlighted that long days at the clinic could be strenuous when they are already feeling unwell, and how they would rather prefer coming in for shorter appointments over different days. These findings together highlighted the need for personalized scheduling considering each patient/care partners’ circumstances and preferences. Patients and care partners found that aligning appointments with their own schedules made their care “less burdensome” and allowed them to maintain a “sense of normalcy.” Some prioritized avoiding rush hour traffic or coming in before or after work; others needed to coordinate with their care partners’ work schedule since the care partner provided transportation and support. They described “a lot of benefit” when the care team was “careful to aggregate appointments,” and clinicians felt clustering appointments was logistically and emotionally beneficial, noting patients are “grateful and almost ecstatic that they save a trip.” However, a small number of respondents indicated that they preferred not stacking appointments on the same day, since appointments could be hours apart anyway—“It is difficult to get all your appointments to line up one after the other”—and it could be “too much” in 1 day for the patient. (5) Having designated schedulers—a one-stop-shop for all scheduling needs—was a recurrent solution proposed. As one patient explained, a designated, individualized care coordinator “would be huge” in maintaining oversight of treatment schedules, streamlining communication, and reducing the emotional toll of repeatedly explaining complex scenarios to multiple staff members. There were also suggestions to (6) ensure only necessary and carefully prepared appointments occur, requiring clinicians and health systems to think critically about which visits are truly needed to advance patient care, and to guarantee everything needed for care is set up when patients arrived, such as making sure laboratory orders were in place and radiology images were obtained, so patients are not “just sitting there waiting.”

### Theme 2: Improved administrative and logistical support

A common view among interviewees was the need to better support patients and care partners with administrative and logistical responsibilities, presented across four subthemes (1–4). Patients and care partners take on significant burden addressing these tasks and noted they would appreciate (1) assistance with health insurance and other costs including medical bills and direct medical costs, and (2) with other logistical tasks such as employment paperwork, FMLA (Family and Medical Leave Act) paperwork, applying for social security, etc. Participants voiced frequent struggles with payers denying coverage for essential care, receiving medical bills, and limited awareness of financial assistance programs. They additionally noted they received limited support from cancer care teams for tasks such as employment challenges (e.g., “job lock”) for example. Patients and care partners did acknowledge the immense relief and gratitude when someone unexpectedly did help them with otherwise seemingly impossible tasks, such as helping apply for a financial grant (“That $1000 was a lifesaver then”) or receive a pharmacy discount coupon (“somebody helped me use the online app and they showed me what to do”).

Empathetically, patients and care partners spontaneously brought up the need for (3) administrative support for clinicians, and how taking these burdens off their plates would lead to downstream benefits for patients/care partners. One care partner noted they felt bad asking busy clinicians to “sign my little piece of paperwork here again.” Clinicians also endorsed being overwhelmed with tasks extraneous to direct patient care, including describing “begging” insurance companies to approve treatments as “the worst toxicity ever that I would not wish on my worst enemy.” Participants recognized that even with well-intentioned clinical teams, (4) current staffing levels are inadequate to help patients and care partners with these types of tasks—that there are not enough, social workers, for example, to help in “the land of fire.” Interviewees proposed a designated staff person in oncology clinics dedicated to administrative aid for patients and clinicians as a potential solution to these shortages, acknowledging the need for health system buy-in. Together these results provide important insights into supporting all stakeholders with logistical and administrative burdens.

### Theme 3: Home-based care when safe, feasible, and preferred

The theme of decreasing time burdens by receiving care in one’s own home when safe, feasible, and clinically appropriate, came up repeatedly and is discussed across three (1–3) subthemes. Participants brough up that both (1) telemedicine appointments, especially for simple check-ins, when patients were usually doing well, and on “maintenance treatment,” and other (2) formal home-based health services, such as with home nursing, physical therapy, and intravenous fluid administration, could decrease travel, parking, and waiting times, while allowing patients to be in the “comfort of my house.” As one participant commented, home-based care was “so much less stressful” and replaced “really, really annoying” clinic visits. Individuals undergoing treatment with 5-fluorouracil-based chemotherapy, that requires a chemotherapy pump disconnect usually on day three, were thrilled to have the option of a home nurse visit to disconnect the chemotherapy, and to not have to go back into the infusion center when the patient “felt crappy” after chemotherapy. In addition to the time savings associated with home-based care, participants recognized additional benefits such as, “mentally and psychologically, there’s a comfort level already,” especially when they established longer-term relationships with home care staff.

Recognizing the complexity of care coverage and delivery, and how much care is dictated by policy and payers, participants across stakeholder groups stated (3) policy reform and systemic support for home-based care was needed to allow patients and care partners to fully harness the time savings for home-based care. A clinician noted that requiring patients to be “homebound” to qualify for certain home-based services under current Medicare policy [24] “made no sense,” and a patient shared their experience with initially being allowed to get shots at home but having to transition to clinic later because “insurance wouldn’t pay.”

### Theme 4: Transparent and empathetic communication of time demands

Patients and care partners noted that they desired (1) information and guidance from their oncology care teams regarding time burdens, and that such information would help set (2) clear expectations about expected time burdens. They also discussed that (3) clinical teams showing interest in addressing these time burdens—even without a direct solution—would be helpful for them. A care partner noted they were told an infusion “would be quick” by their oncologist, but the infusion appointment was then booked for 3 h and ultimately took 5 h. There was a desire for “transparency” and “honesty” regarding what to expect, especially when first diagnosed and starting treatment.

Specifically, participants felt that (4) communicating about time burdens using objective measures (e.g.,: a 5-h infusion) instead of more frequently used subjective qualifiers (e.g.,: “quick” as mentioned above) would be useful in communicating expected time burdens and could be used to help with (5) informed decision-making around time-intensive treatments. A clinician reported that a patient of theirs (unrelated to and unaware of the study) had stated “I’m not coming in on that day, that’s my only home day,” highlighting the intuitiveness of a previously described contact day vs home day measure. Participants recognized that time toxicity is only one factor among several (such as life context, e.g., a young parent, financial burdens) when deciding to pursue treatments with marginal survival benefits but felt that information on expected time toxicity “would be a really important piece of information” and would make “a world of difference.” However, clinicians also highlighted that they are often unaware of exactly how long patients are needing to spend in the clinic to receive an infusion, emphasizing the importance of (6) availability of objective data about time burdens for clinicians to use in counseling patients. Participants shared that a definition of time toxicity such as “contact days,” as used in previous work, could be one helpful tool in measuring burdens to facilitate increased objectivity. Clinicians reflected that discussing time burdens in this way should be “obvious’’ but “when it's not at the forefront… we are less likely to make small changes that would make a meaningful difference for our patients.”

### Theme 5: Innovations in care models and delivery

All three participant groups discussed how research and innovation in cancer treatment and care delivery have improved their experienced time toxicity. Innovations, when appropriate, such as (1) shorter treatment courses, (2) oral treatment options, (3) less frequent treatment schedules, and (4) faster formulations such as subcutaneous injections instead of long infusions reduced the amount of time patients were spending in clinic, in waiting rooms, and in travel to and from visits. One patient detailed getting “hours of my day back” because of a switch from an hours-long infusion to a 5-min injection, while another was able to stop chemotherapy 3 months early because a new trial demonstrated 6 months was “just as good” as three (the IDEA trial in colon cancer). Additional benefits of oral medications such as taking them in the “comfort” of home instead of clinic and availability of mail order pharmacies reduced time toxicity for patients and their care partners. These novel treatment modalities provide additional choice and associated sense of control for individuals undergoing cancer treatment.

On the clinician side, they emphasized the piloting of time saving benefits of (5) digital communication technologies to assist with paperwork completion and artificial intelligence assistance in documentation. Both clinicians and patients discussed how reducing the time burden of non-patient facing tasks for clinicians will allow for additional time in the clinic room, thereby improving the patient experience.

## Discussion

This qualitative study of patients, care partners, and clinicians explored solutions to decrease the time burdens associated with cancer care. Participants highlighted personalized scheduling of ambulatory care considering their preferences, support for logistical and administrative tasks such as help with understanding medical insurance and bills, and the option for home-based care when safe and feasible as key strategies. They additionally indicated the desire for clear, objective communication from their cancer care team regarding expected time burdens, potentially using the contact days measure, both to set expectations and to factor into decision-making when pursuing time-intensive treatments. Moreover, innovations in care design and delivery—such as shorter total treatment courses and faster treatment delivery—could decrease time burdens. These perspectives from persons with lived or professional experience with cancer provide a vast and informative array of potential solutions as the concept of time toxicity matures, and health systems and policymakers look to implement solutions.

Modern oncology care is largely delivered in the ambulatory setting, and attending frequent, uncoordinated appointments, with the extraneous travel, parking, and wait times, adds tremendous burden [25, 26]. In one study, the median round trip distance for over 6000 U.S. commercial beneficiaries with pancreatic cancer receiving FOLFIRINOX chemotherapy was 196 miles [27]—providing care closer to home represents an obvious solution [28]. Older adults with advanced stage cancer receiving chemotherapy spend a fifth of their days with health care contact, and most of these are ambulatory contact days [9]. Over two-thirds of ambulatory contact days have just one service performed [9, 29], highlighting opportunities to better coordinate ambulatory appointments. Bunching appointments together on a single day was suggested as a solution by several participants and is intuitive. This may be particularly relevant just after diagnosis, when patients typically undergo intensive diagnostic evaluation and see multiple specialists and may spend 40% of days in the first couple of months around diagnosis attending appointments [21, 22]. Opportunity also exists to ensure that labs, imaging, infusions, and clinic visits are more streamlined, limiting the amount of time patients spend in waiting rooms or in transport. Single-day multidisciplinary clinics, where multiple specialists evaluate the patient on the same day, with a clear plan at the end, are one solution [30]. However, highlighting the complexity of the problem, not all participants preferred to bunch appointments on the same day—patient and care partner preferences regarding this may also differ, as also previously demonstrated when asked about how they viewed home-based care [2]. Patients valued longitudinal relationships with the scheduling team and appreciated when appointments could be adjusted around their own schedule. Based in part on the current study’s feedback, clinicians at our cancer center are alerted 2 days prior to lab appointments if lab orders are not in place for an upcoming appointment. Prior work has demonstrated that having “orders in place” is key to decreasing patient wait times [31] which participants in this study also agreed with, noting that time spent waiting between clustered appointments reduces the benefit of grouping them in the first place. Individual care coordinators and navigators could assist in taking these personal preferences into account.

Patients and care partners expressed that support with administrative and logistical tasks, such as dealing with insurance paperwork, handling medical bills, and applying for social security, for example, would save them time [2, 12, 32, 33]. Financial, administrative, and time burdens can overlap and amplify associated distress [13], and current oncology care teams, even if well-intentioned, are undertrained, under-resourced, and understaffed to help vulnerable and overwhelmed patients and care partners with these burdens. Proactive support with navigation and legal support may help address these, as being studied in trials (e.g., NCT06475664, Supplementary Table 3). Innovations in site and mode of delivery of care also have the potential for time savings, with telemedicine showing decreased time burdens when compared to in-person visits [34], translating into estimated average cost savings of $147 to $186 per visit for patients [35], and recent work has highlighted patients broadly support diverse visit types conducted through telemedicine, including clinical trial consenting [36]. Home phlebotomy, hospital-at-home and supportive care-at-home programs are already established, and trials (e.g., NCT05969860) are testing home-based and telemedicine-based care approaches [37–40]. However, it is also important to highlight that, while in this paper participants discussed home-based care through a solutions-focused lens, our previous qualitative study delving more specifically into care at home detailed that it can, in certain cases, worsen burdens particularly on care partners [2].

Similar to the financial toxicity literature [41], we found that patients expressed a desire for conversations about time toxicity initiated by their oncology care team early in the treatment course. This information would help set realistic expectations about care and would factor into patients’ decisions about whether to pursue treatments, although time burdens are only one factor in decision-making. In separate work, 47% of patients unprompted brought up the time burdens of care as an essential factor in deciding whether they would pursue treatments with marginal survival benefits [42]—this was highlighted in the current work as well, with patients and care partners bringing up the importance of time considerations in shared decision making. However, clinicians need additional resources about the time burdens of the treatments they are prescribing to share this desired, objective information with patients. Resources with estimates on duration of infusions, such as eviQ in Australia, incorporation of data collection around time burdens in clinical trials, and the utilization of objective, patient-centered measures such as “contact days” are all potential solutions to address the desire for more objective communication regarding time burdens [43]. Participants also highlighted innovations in care, such as shorter treatment courses (e.g., hypofractionated radiation), oral treatment options (e.g., capecitabine), less frequent options (e.g., every 3-month zoledronic acid when appropriate) and schedules (e.g., less frequent dosing for carfilzomib), and faster formulations (e.g., subcutaneous injections) [44], as time savings. Innovative technology-based interventions, such as e-triage through text messaging systems to bypass unnecessary clinician visits prior to immunotherapy infusions [14], or shorter-duration outpatient monitoring instead of multi-day hospitalizations [45], represent on-the-ground applications and solutions.

Across all themes, we noted the critical role of policies and coverage/reimbursement decisions in driving care patterns and thus determining the feasibility and sustainability of certain solutions to decrease time burdens. The Centers for Medicare & Medicaid Services finalized Principal Illness Navigation (PIN) Services codes in 2024 that can support cancer centers’ efforts to provide formal navigation support to patients, and training and credentialing programs such as the American Cancer Society Leadership in Oncology Navigation can help build a trained workforce to implement [46, 47]. While extended to September 2025, the future of telemedicine is uncertain, and home-based care faces similar issues.

This study has several limitations including aspects of participant characteristics and its single center location. Specifically, patient selection was limited to individuals with advanced gastrointestinal cancers that underwent cancer-directed therapy. These patients and associated care partners likely have a different perspective on time burdens and goals of care compared to those with other diagnoses or who may have opted for a more comfort-focused approach. However, gastrointestinal cancers do overall represent a wide range of clinical experience. All clinicians, while varied in their job titles, work at an NCI-Designated Cancer Center and a major academic institution, likely with different experiences than providers in more community or rural practice. Patients also all received care at this NCI-designated facility, limiting perspectives. However, some patients were co-managed with other health systems or had transferred care from elsewhere, allowing for diversity in experiences.

## Conclusions

This study adds to a growing body of literature on time toxicity, contributing perspectives from key stakeholders including patients, their care partners, and clinicians on how to address time toxicity in cancer care. Proposed solutions include schedulers designated to individual patients, considering their needs and preferences and ensuring all needed information is in place to avoid unnecessary visits through systematic means, such as mandated lab orders and EHR alerts. Prioritization of assistance for logistic and administrative burdens would take these tasks off care partners’ plates, allowing them to spend more time with their loved one. Clinicians should prioritize research discerning how to reduce treatment-related time burdens through innovation like oral and subcutaneous therapies, shorter treatment courses, and less frequent appointments while maintaining the highest levels of quality, safety, tolerability, and efficacy, and addressing liability concerns. This is particularly crucial in the context of toxic cancer treatments and when considering health equity, especially for older adults and other vulnerable populations; ensuring that we are not equating a lack of access to health care as superior from a time toxicity standpoint. Additionally, more robust data collection is needed to quantify actual time burdens of cancer therapies and inform the development of patient-centered care delivery models.

## Supplementary Information

Below is the link to the electronic supplementary material.

Supplementary Material 1 (DOCX 34.1 KB)

## Data Availability

No datasets were generated or analysed during the current study.
