# Supplementary material for: Patient-, care partner-, and clinician-proposed solutions to address the time toxicity of cancer care
Source: Support Care Cancer. 2025 Oct 18;33(11):965. doi: 10.1007/s00520-025-09954-0 (PMC12534262; doi:10.1007/s00520-025-09954-0)
Supplement: Supplementary file 1 — Supplementary Material 1 (DOCX 34.1 KB) [file 520_2025_9954_MOESM1_ESM.docx]

**Supplementary Material:**

**Patient, Care Partner, and Clinician Proposed Solutions to Address the Time Toxicity of Oncology Care**

**Whitney V. Johnson et al.**

**Supportive Care in cancer**

**Corresponding author:**

Arjun Gupta, MD

Assistant Professor,

Division of Hematology, Oncology, and Transplantation,

University of Minnesota

516 Delaware Street SE, MMC 480, PWB 14-100, Minneapolis, MN 55455

Email: [arjgupta@umn.edu](mailto:arjgupta@umn.edu)

The Consolidated criteria for reporting qualitative research (COREQ) checklist and a portion of the interview guide have been presented in prior work from the same cohort of participants.^1^

1. Gupta A, Johnson WV, Henderson NL, et al. Patient, Caregiver, and Clinician Perspectives on the Time Burdens of Cancer Care. *JAMA Netw Open*. Nov 4 2024;7(11):e2447649. doi:10.1001/jamanetworkopen.2024.47649

**Supplementary Table 1:** Consolidated criteria for reporting qualitative research (COREQ) checklist ^1^

| **Topic** | **Item No.** | **Guide Questions** | **Description** |
| --- | --- | --- | --- |
| **Domain 1: Research team and reflexivity** | | | |
| Interviewer/facilitator | 1 | Which author/s conducted the interview or focus group? | Interviews were conducted by 1 trained interviewer (OOO). |
| Credentials | 2 | What were the researcher’s credentials? E.g., PhD, MD | A Master’s degree in Higher Education Leadership. |
| Occupation | 3 | What was their occupation at the time of the study? | Enrolled in a Ph.D. program at the University of Minnesota. |
| Gender | 4 | Was the researcher male or female? | Male. |
| Experience and training | 5 | What experience or training did the researcher have? | Over ten years of experience in qualitative research including conducting interviews, coding data, and reporting. |
| Relationship established | 6 | Was a relationship established prior to study commencement? | No interviewers had pre-existing relationships with participants. |
| Participant knowledge of the interviewer | 7 | What did the participants know about the researcher? e.g., personal goals, reasons for doing the research | Participants had no prior knowledge regarding the interviewer. |
| Interviewer characteristics | 8 | What characteristics were reported about the interviewer/facilitator? e.g., Bias, assumptions, reasons and interests in the research topic | The interviewer was involved in a variety of clinical patient oriented research studies. |
| **Domain 2: Study design** | | | |
| Methodological orientation and Theory | 9 | What methodological orientation was stated to underpin the study? e.g.  grounded theory, discourse analysis, ethnography, phenomenology, content analysis | We used grounded theory and a constant comparative methods to conduct an inductive thematic analysis. |
| Sampling | 10 | How were participants selected? e.g., purposive, convenience, consecutive, snowball | We used purposive and criterion sampling method to recruit a demographically diverse study population, striving for representation across primary gastrointestinal cancer sites, age, and gender, and among clinicians, by primary role. |
| Method of approach | 11 | How were participants approached? e.g., face-to-face, telephone, mail, email | We identified potentially eligible patients and care partners during clinic visits; they were approached by a study coordinator unrelated to the treatment team in-person. Potential clinicians were approached by the study coordinators in clinic in-person. |
| Sample size | 12 | How many participants were in the study? | We aimed to interview approximately 15-20 participants per group with no fixed sample size, aiming for saturation. We eventually included 47 participants (16 patients, 15 care partners, and 16 clinicians). |
| Non-participation | 13 | How many people refused to participate or dropped out? Reasons? | Two patients declined participation due to lack of time, and interviews could not be scheduled among 2 clinicians due to inability to schedule.  No enrolled participants ended their interview early or dropped out of the study. |
| Setting of data collection | 14 | Where was the data collected? e.g., home, clinic, workplace | Participants chose the interview format [video via Zoom (n=21), phone (n=16), and in-person in private rooms at the Clinic (n=10)], and scheduling per their convenience and preference. In-person interviews occurred in private rooms in a location separate from the typical clinical area. |
| Presence of non-participants | 15 | Was anyone else present besides the participants and researchers? | Non-participants were not present for the interview. Patients and care partners (a dyad) had the opportunity to participate separately or together in the interview based on preference (for 2 dyads). |
| Description of sample | 16 | What are the important characteristics of the sample? e.g., demographic data. | Refer to Results, Table 1 and supplementary material for participant demographics. |
| Interview guide | 17 | Were questions, prompts, guides provided by the authors? Was it pilot tested? | An inter-disciplinary study team including patient and care partner advocates, researchers with expertise in qualitative methods and treatment burden, and oncologists, initially developed the interview guide, after a literature review on the time burdens of cancer care. This was refined after the pilot interviews. |
| Repeat interviews | 18 | Were repeat interviews carried out? If yes, how many? | Participation ended once the initial interview was complete with no additional follow-up. There were no repeat interviews carried out, meaning that each interview represents a unique participant. |
| Audio/visual recording | 19 | Did the research use audio or visual recording to collect the data? | Interviews were audio-recorded, transcribed and reviewed for interview quality. |
| Field notes | 20 | Were field notes made during and/or after the interview or focus group? | Field notes were recorded during and after the interviews to provide additional context and information regarding the circumstances of each interview. |
| Duration | 21 | What was the duration of the inter views or focus group? | On average, the interviews lasted 45 minutes. |
| Data saturation | 22 | Was data saturation discussed? | Once the interviewers perceived that data saturation was reached, the research team decided to conclude scheduling additional interviews and moved forward with the formal data analysis stage. |
| Transcripts returned | 23 | Were transcripts returned to participants for comment and/or correction? | Participation ended once the interview was complete with no transcript return. |
| *Data analysis* | | | |
| Number of data coders | 24 | How many data coders coded the data? | For primary analysis, an initial coding schema was developed by two investigators. |
| Description of the coding tree | 25 | Did authors provide a description of the coding tree? | A codebook, or coding tree, was created to clearly organize and define the most relevant codes and themes. |
| Derivation of themes | 26 | Were themes identified in advance or derived from the data? | Themes were derived from the data. |
| Software | 27 | What software, if applicable, was used to manage the data? | NVivo software. |
| Participant checking | 28 | Did participants provide feedback on the findings? | Participant checking occurred in real-time during the interviews as a validation technique. As participants responded to questions, the interviewers would state their immediate interpretations to validate or confirm that their interpretation of what the participant has said aligns with the participant’s intention. |
| Quotations presented | 29 | Were participant quotations presented to illustrate the themes/findings?  Was each quotation identified? e.g., participant number | Illustrative quotations are presented in the Results and in Table 2 with appropriate participant identification. |
| Data and findings consistent | 30 | Was there consistency between the data presented and the findings? | There was consistency between the interview data and the findings presented. |
| Clarity of major themes | 31 | Were major themes clearly presented in the findings? | Major themes from our analysis are reported in the Results section. |
| Clarity of minor themes | 32 | Is there a description of diverse cases or discussion of minor themes? | Subthemes are clearly described and discussed. |

**Supplementary Table 2:** Patient Interview Guide ^1^

(similar interview guides were used for care partners and clinicians)

**Introduction**

Hello, my name is [______], and I am a [JOB TITLE/ROLE] from the University of Minnesota. I’m currently working on a project studying the time burdens of cancer treatment and their effects on patients and their families. While there are some overlaps, the cancer treatment experience is different for everyone, so we’re talking with a number of different patients about what the process was like for them.

The interview should take about 30-45 minutes and as we go through the questions, please know that your participation is completely voluntary and you may choose not to answer to end the interview at any time.

If it’s okay with you, I would like to record our interview, so that I can focus on our conversation now but still compare your experiences to other people that I talk to. The only people who have access to the recording will be our trained research staff and if we use the information you share in presentations or publications, it will be anonymous. Is it okay if I record?

**Part 1:**

1. We are interested in exploring the entire process of your experience with cancer care. Starting at the beginning when you first met with an oncologist after being diagnosed, were there any time burdens you faced before starting cancer treatment?

Was there anything you had to do to prepare for this first meeting?

Were you spending time thinking about treatment or doing research on it?

1. Moving on to the time that you began receiving treatment, what have been the time burdens that you generally face?
2. Among the different time burdens mentioned, which ones are the most burdensome for you?

- What makes this burdensome?

- Is there anything that helps lessen the burden?

- Is there anything that makes it worse?

- Is there anything that could help but hasn’t been done?

1. With cancer care, there are many things that are scheduled ahead of time but then there are also unscheduled things that occur. Can you talk about some of the unscheduled things that took up time during treatment?

- Such as dealing with side effects of treatment, time spent on phone with insurance, etc.

1. With so much time sent on treatment, many patients end up missing important work or life events because they were busy with cancer care. Have you experienced this, and if so, can you tell me about it?
2. We are also curious about care partners such as friends and family who support you. In what ways do you think your care partners’ time has been affected in relation to your treatment?

**Part 2:**

1. In what ways have the cancer care team helped decrease the time burdens that you and your care partners face?

- What more could be done?

1. What other suggestions would you have to reduce the time burdens faced by you or your care partners?
2. What are your thoughts on using virtual visits in comparison to in-person visits as a way to reduce time burdens? How about home-based care?

- Does this help? Are there any drawbacks?

1. Since you have gone through the treatment process, if you knew back then at the beginning what you know now about how much of a time commitment your treatment would be, do you think that would have changed your mind about which treatment you proceeded with?

5. To what extent do you think time commitments should be factored into treatment decision making?

6. Is there anything else you would like to add? Anything you feel was missed in this discussion?

**End of Interview**

**Supplementary Table 3:** Examples of actionable solutions to address time burdens of cancer care

| **Group primarily benefiting** | **Actions implemented and being assessed** |
| --- | --- |
| **Patients and Care Partners** | Proactive administrative support including upfront financial and legal resources (as being studied in a current clinical trial - NCT06475664). |
| **Clinicians** | Adoption of DocuSign electronic signature platform to reduce paperwork associated time burdens, allowing clinicians more time to spend with patients. |
| **Patients, Care Partners, and Clinicians** | Alerting clinicians two days prior to patient lab appointments if lab orders are not in place - reducing patient wait times and need for rescheduled visits in addition to data available to review in clinic for more effective time with clinicians. |
